# Evaluation of the Effectiveness of Combined Treatment with Intravaginal Diazepam and Pelvic Floor Rehabilitation in Patients with Vulvodynia by Ultrasound Monitoring of Biometric Parameters of Pelvic Muscles: A Pilot Study

**DOI:** 10.3390/diseases12080174

**Published:** 2024-08-01

**Authors:** Lucia Merlino, Enrico Ciminello, Agnese Immacolata Volpicelli, Stefano Tillier, Marianna Francesca Pasquali, Mattia Dominoni, Barbara Gardella, Roberto Senatori, Barbara Dionisi, Maria Grazia Piccioni

**Affiliations:** 1 Department of Medical-Surgical Sciences and Biotechnologies, Sapienza University of Rome, 00161 Rome, Italy; lucia.merlino@uniroma1.it (L.M.); mariagrazia.piccioni@uniroma1.it (M.G.P.); 2 Italian National Institute of Health, 00161 Rome, Italy; enrico.ciminello@iss.it; 3 School of Gynecology and Obstetrics, University of Eastern Piedmont, 28100 Novara, Italystefano.tillier@gmail.com (S.T.); 4 Department of Clinical, Surgical, Diagnostic and Pediatric Sciences, University of Pavia, 27100 Pavia, Italy; mariannepasquali@gmail.com (M.F.P.); barbara.gardella@gmail.com (B.G.); 5 Department of Obstetrics and Gynecology, IRCCS Fondazione Policlinico San Matteo, 27100 Pavia, Italy; 6 Vulvar Pathology Study Center, 00161 Roma, Italy; robertosenatori@gmail.com (R.S.); barbaradionisi@gmail.com (B.D.)

**Keywords:** vulvodynia, pelvis floor, rehabilitation, pain

## Abstract

(1) Background: Vulvodynia is characterized by vulvar pain for at least three months and may have related variables, one of these being pelvic floor hypertonus. The purpose of this study was to compare the therapeutic effectiveness of two weekly sessions of pelvic floor rehabilitation and 5 mg of vaginal diazepam daily vs. pelvic floor rehabilitation alone in individuals with vulvodynia. (2) Methods: A single-center, not-blind, randomized study enrolled 20 vulvodynic patients: A total of 10 were treated with dual therapy (intravaginal diazepam and pelvic floor rehabilitation), and 10 were treated with only pelvic floor rehabilitation. All of them underwent a pelvic floor ultrasound examination and VAS pain and Marinoff scale assessments before the beginning of therapy as well as three and six months later. (3) Results: The elevator plate angle ranged from 8.2 to 9.55 (*p* = 0.0005), hiatal area diameter ranged from 1.277 to 1.482 (*p* = 0.0002), levator symphysis distance ranged from 3.88 to 4.098 (*p* = 0.006), anorectal angle ranged from 121.9 to 125.49 (*p* = 0.006), Marinoff scale ranged from 2.3 to 1.4 (*p* = 0.009), and VAS scale ranged from 5.8 to 2.8 (*p* < 0.001). (4) Conclusions: This pilot study demonstrates that the suggested treatment improves the hypertonicity of the pelvic floor, as measured by ultrasound parameters, correlating with a reduction in symptomatology.

## 1. Introduction

Vulvodynia is a persistent idiopathic vulvar pathology characterized by the presence of vulvar pain for at least three months. Since 2015, the International Society for the Study of Vaginal Disease (ISSVD) has recognized it as a distinct entity from vaginal pain syndrome [1]. Its prevalence ranges from 3 to 15%; however, it is challenging to assess because of its variety of presentation, frequent correlation with other comorbidities, and neuromodulation through anxiety status or estrogen insufficiency [2,3].

Vulvodynia can occur in various forms: it can be localized to one area, generalized to numerous ones, or mixed (localized and generalized). Pain can occur spontaneously, be provoked (by intercourse with vaginal penetration, insertion of tampons, or contact with the vulva), or be combined (provoked and spontaneous). The pattern of pain can be either rhythmic or intermittent, brief or prolonged, continuous or constant. The two most prevalent subtypes are provoked vestibulodynia (localized vulvodynia at the vulval vestibule in which physical contact generates pain) and generalized spontaneous vulvodynia (where pain is widespread and unprovoked) [4].

The most common clinical presentation is dyspareunia. However, other symptoms often coexist, like neuropathic pain symptoms (allodynia, burning, paresthesia), discomfort during or after urination or defecation, changes in the distribution or intensity of pain, concurrent medical disorders, and pelvic floor trigger point pain, that may suggest a central sensitization.

Due to the stigma associated with female pain and the sexual area, patients are reluctant to disclose their symptoms to clinicians, and so vulvodynia is still understudied. Furthermore, due to the complexity of the disease, the exact etiology is still unknown, even if a multifactorial origin seems increasingly clear. In particular, it has been shown that there is a multiplication of nerve fibers and persistent inflammation in painful areas. Nevertheless, it is still unclear how central sensitization relates to chronic pelvic pain and how people with this illness would react to the standard therapy for this pain in comparison to those who are not afflicted by it.

In this clinical picture, the involvement of the pelvic floor muscles has been established in the etiology of this disease. In fact, it has been observed that 80–90% of women suffering from vulvodynia present pelvic floor hypertonus [3]. In view of this, a growing number of studies are present in the literature that analyze therapeutic proposals based on actively working on the muscular component.

The aim of this pilot study was to evaluate the therapeutic efficacy of the combination of diazepam 5 mg administered vaginally once a day with two sessions per week of rehabilitation of the pelvic floor muscles vs. pelvic floor muscle rehabilitation alone in patients suffering from clinically assessed vulvodynia.

## 2. Materials and Methods

The Ethics Committee of La Sapienza University of Rome approved the experimental protocol, and written consent was obtained from all participating patients. A total of 32 patients were initially screened, and 20 patients were enrolled in this monocentric, not-blind, randomized trial in a 1:1 ratio. A total of 12 patients were excluded from this study before the randomization process because of their choice not to use vaginal therapy. After enrollment, the patients were randomly assigned to study groups according to a pre-designed schedule and were allocated with equal probability to either intravaginal diazepam and pelvic floor rehabilitation or pelvic floor rehabilitation only. The drug administered was revealed to the patients.

Women who came to our outpatient clinic, Vulvar Pathology Study Center, from 1 October 2022 to 30 November 2022 were followed until February 2023 and May 2023. This study was conducted in accordance with the Declaration of Helsinki and approved by the Institutional Review Board of the University of La Sapienza in Rome. The patients voluntarily complied with this study, and the recruitment took place during the first gynecological examination. A complete gynecologic examination and clinical evaluation were performed by the referring gynecologist.

The exclusion criteria were the following: autoimmune diseases, neurological disease, neuropathies, menopause, continuous estrogen amenorrhea, infectious diseases of the lower tract, sexually transmitted diseases such as *C. Trachomatis* or *N. Gonorrhea*, allergies to benzodiazepines, and pregnancy.

We included sexually active patients aged between 18 and 45 years with vulvar pain for at least three months who were evaluated with the features listed below.

The impact and, thus, the effectiveness of the proposed treatment were assessed by analyzing the changes in ultrasound biometric parameters and, secondarily, in clinical parameters at time 0 and 6 months after treatment:

- Ultrasound biometric parameters: symphysis: anus elevator distance, anorectal angle, and diameter of the hiatal area, all assessed trans-labial;
- Clinical parameters: dyspareunia assessed by the Marinoff scale; vulvar pain assessed by the visual analogic scale (VAS);
- The diagnostic approach used is based on that proposed by Bergeron [5].

During the first visit, a complete pathological history was taken, both remote and forthcoming, and the patient was asked to assess vulvar pain by means of the VAS and to assess the degree of self-referred dyspareunia using the Marinoff scale: 0 (absence of pain), 1 (pain that does not interfere with the frequency of intercourse even if it causes discomfort), 2 (pain that occasionally prevents intercourse), and 3 (pain that always prevents intercourse).

The gynecological examination was then performed, starting with the inspection, to assess the presence of vulvar erythema, inflammatory dermatological pathologies, or vulvovaginal atrophy that may mimic vulvodynia-like symptoms.

Afterwards, the cervix and vaginal walls were assessed through specular examination. A secretion sample was collected, treated with KOH, and observed under the wet-mount microscope to exclude the presence of hyphae; another sample was sent to the microbiology laboratory to confirm the absence or presence of Candida infection, a very common picture in these women. In the event of culture positivity, the patient was then treated with itraconazole. In addition, we excluded sexually transmitted diseases from the laboratory evaluation of the vaginal swab. Eventually, a test was performed to assess the vaginal pH.

On the vulva and vaginal introitus, the cotton swab test was conducted, starting with the most peripheral component, i.e., the mount of Venus, the medial aspect of the proximal part of the thighs, to make the patient familiar with the examination, and then moving on to the various vulvar structures in order to understand whether hypersensitivity in this area was present.

With positivity to the latter test, excluding the inflammatory, infectious component, and a picture of vulvovaginal atrophy, a diagnosis of vulvodynia could be made.

Afterwards, the selected vulvodynic patients underwent trans-labial ultrasound with a 4D convex probe with an empty bladder. This choice is motivated by the need to seek an evaluation method that is as objective as possible and that allows for an actual improvement in pelvic floor hypertonus to be assessed in numerical terms. Although ultrasound is an operator-dependent method, in this context, it is minimally invasive and, especially, painless since it does not require vaginal penetration, thus eliminating the potential involuntary contraction of the pelvic muscles [6].

The parameters that have been calculated are the following:

- Resting symphysis-elevator distance: distance from the lower posterior limit of the anorectal junction;
- Anorectal angle at rest: the angle is measured at the confluence of the drooping lines on the longitudinal axis of the anal canal and the posteroinferior margin of the rectal wall;
- Elevator plate angle: the angle is obtained by the intersection of the horizontal reference line embedded at the level of the pubic symphysis at the crux of the anorectal angle;

Diameter of the hiatal area: the hiatal area is bound laterally by the pubo-rectal muscle, anteriorly by the pubic symphysis, and posteriorly by the inferior pubic branch.

**Figure 1:**
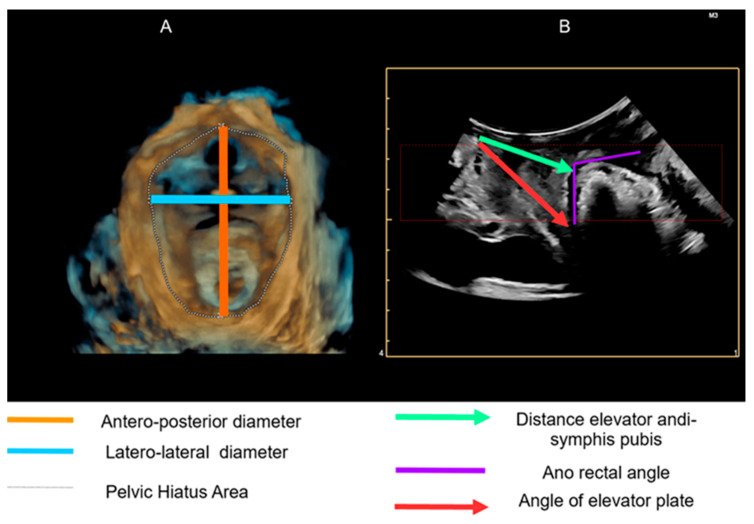
Evaluation of the pelvic floor hypertonus through objective measurement of selected parameters using trans-labial ultrasound with a 4D Convex Probe. (**A**): 3D reproduction; (**B**): 2D reproduction.

These parameters are not the result of a random choice but rather weighted, taking into account the present literature, and are illustrated in Figure 1 [6,7,8].

Once these parameters were obtained, the patients were divided into two groups of 10 people in a randomized manner, and the first group was prescribed two weekly pelvic floor rehabilitation sessions and the use of diazepam 5 mg intravaginally every evening. The control group was prescribed only two weekly pelvic floor rehabilitation sessions.

The choice of administering diazepam intravaginally led to the need for a galenic formulation, the combination chosen being the following:

- Excipients: hydrophilic excipients, as they are better suited to the vaginal environment. The chosen mixture is based on PEG (polyethylene glycol) 400 and PEG 4000, 7% and 40%, respectively;
- Molds: vaginal ovules containing 12 mg; Active ingredient: diazepam. The composition of a single ovule contains 0.005 g diazepam, 1.5015 g PEG 400, and 1.01 g PEG 4000.

Before deciding to proceed with this type of treatment, a careful literature review was conducted to analyze the presence of side-effects and study the pharmacokinetics and pharmacodynamics of intravaginal administration. With regard to pharmacokinetics, in a study conducted on a sample of women who regularly took at least one dose of diazepam 10 mg vaginally, it was observed that the serum concentration of diazepam was within the normal range [9]. However, it should be noted that the time interval between the last administration of the drug and the blood sample is not standardized. Concerning the side-effects, the only one reported is drowsiness. Two studies had 33% and 10% of women, respectively, experiencing this side-effect; however, administering the drug in the evening seems to reduce its presentation [9,10].

As for pelvic floor rehabilitation, the physiotherapist first performed a detailed analysis through the following:

- Pelvic floor evaluation to assess the presence of hypertonus;
- Assessment of the basin;
- Evaluation of the column;
- Functionality and muscle symmetry;
- Capacity for synchronous contraction.

On the basis of this assessment, the treatment plan was set up, based on the following:

- Pelvic floor activation exercises;
- Respiratory coordination exercises;
- Leg coordination exercises;
- Pelvic floor coordination exercises;
- External massage of the pelvic musculature;
- Pelvic floor muscle stretching;
- Biofeedback;
- Transcutaneous electrical nerve stimulation (TENS) with a vaginal probe and external electrodes;
- Training for muscle-relaxing self-postures and use of a hot water bottle for continuing physiotherapy at home.

### Statistical Analysis

The data are presented in terms of the mean (standard deviation). The statistical significance of variations in eco-parameters between the time before and after the treatment is investigated via the Wilcoxon test for paired samples. The statistical significance of the differences in variations between case arm and control arm is tested via the Mann–Whitney test. The association of the treatment with the Marinoff score and VAS is explored by linear models, both unadjusted and adjusted, for the starting clinical condition provided by the eco-parameters. The significance threshold for *p*-values is fixed equal to 0.05. The statistical analysis is performed by R version 4.2.2 (31 October 2022 ucrt)—“Innocent and Trusting”.

## 3. Results

Twenty patients were enrolled, with an average age at the first visit of 22.6 years and average BMI of 19.34, who had no evidence of previous pathology and, so, were not taking any known therapy.

The ultrasound parameters and the considered scores for pain are summarized in Table 1. The observed variations between pre- and post-treatment are significant for elevator plate angle and hiatal area diameter for the case arm (*p* < 0.01), while they are all significant in the control arm. Both the Marinoff score and VAS decreased significantly after treatment in the case arm (*p* = 0.01); on the other hand, the Marinoff score increased significantly in the control arm (*p* = 0.03), while the VAS score increased without a statistical significance in this group (*p* = 0.06).

In the case arm, an increase in all US parameters and a decrease in both pain scores were observed, while the control group always showed a decrease in US parameters and an increase in the pain scores. The differences in variations before and after treatment are significant in all observed eco-measures and in both pain scores (Table 2).

According to both unadjusted and adjusted models, patients receiving combined treatment had a statistically significant better response in terms of both the Marinoff score (Table 3) and VAS (Table 4). Indeed, as observed also in Table 2, both measures decreased after treatment in the case arm and increased in the control arm.

## 4. Discussion

Hypertonus is present in 80–90% of vulvodynic patients and has an important clinical role in the evolution of this pathology. Myofibril contraction and relaxation mechanisms are compromised in the long-term, causing pelvic floor muscle (PFM) stress. It has previously been demonstrated that impaired biomechanics affect PFM function during contraction, which is why the important role of pelvic floor rehabilitation in the treatment of vulvodynia is well-known in the literature [3,7]. PFM hypertonicity can lead to a variety of dysfunctional outcomes, the most commonly mentioned of which are increased sexual pain, pain at vaginal insertion of any kind, and impaired PFM contraction and relaxation.

The primary line of treatment for vulvodynia is physical therapy, which aims to restore PFM function and breaks the cycle of vulvodynia symptoms. Physical therapy techniques allow for the treatment of vulvar pain and dyspareunia in women with vulvodynia. The key objective of this strategy is to address hypertonicity and restore normal muscle, which involves both increasing the ability to contract and relax muscles as well as reorganizing myofibrils made up of actin and myosin [8,9,10].

The results of our preliminary data demonstrated that patients receiving combined treatment responded statistically significantly better in terms of both the VAS and the Marinoff score, according to both unadjusted and adjusted models. In fact, following treatment, all measures rise in the control group and diminish in the case arm. In addition, our clinical outcomes are in accordance with the results presented by Gentilcore-Saunier et al. [8]. The authors also reported that, for women with vulvodynia, following a physical treatment regimen that included stretching and PFM exercises had decreased hypertonicity on electromyography and overall pain intensity ratings.

Analyzing the relationship between physical therapy and pelvic floor ultrasound parameters, our results showed that, in line with evidence from the literature, the case arm showed an increase in all US parameters and a decrease in both pain scores, whereas the control group consistently displayed an increase in pain scores and a decrease in eco-parameters. Thibault-Gagnon et al. [7] examined PFM voluntary activities using a 3D ultrasound examination and showed that women with induced vestibulodynia significantly reduced biometric parameters, such as hiatal size and left–right width of the levator hiatus, during the Valsalva manoeuvre as compared to controls. This result emphasizes the PFM hypertonicity-related lack of coordination of PFM function in vulvodynia-affected women.

Morin et al. [6] conducted a study to analyze the morphometry of the pelvic floor in women with and without vulvodynia and found that, in women with vulvodynia, both the size of the hiatal area of the elevator and the anorectal angle are significantly smaller than in patients without vulvodynia. These patients also experienced a decrease in strength and an increase in muscle tone.

Bardin et al. also present a study aimed at analyzing the change in biometric parameters in patients with vulvodynia, randomly assigned to kinesiotherapy and amitriptyline or amitriptyline alone in the control group. In Bardin’s paper, a significant increase in the size of the anorectal angle and the elevator symphysis distance is shown. These results are evidence in favor of the fact that working on the pelvic floor leads to a functional anatomical improvement of the pelvic district, and this leads to an improvement in the clinical picture of patients [11].

By analyzing the validity of pelvic floor rehabilitation and the potential usefulness of intravaginal diazepam, Murina et al. [12] demonstrated that VAS pain scores dropped significantly (*p* = 0.01) from baseline values of 7.5 and 7.2 for the diazepam and control group (placebo administration), respectively, to 4.7 and 4.3; however, this difference was not statistically significant. The diazepam group’s Marinoff dyspareunia ratings differed significantly (*p* = 0.05) from patients in the control group. The diazepam group had a considerably better ability to relax the PFM following contraction (difference between maximal contraction and rest tone) in comparison to the control group. Given that physical therapy and vaginal diazepam work differently to treat vulvar pain and dyspareunia, women with vulvodynia may benefit from a combination of these two therapies. The low risk of adverse effects associated with intravaginal diazepam use was reassured by Carrico et al. [13] and Rogalski et al. [14] and who showed that, after one month of therapy, daily dosages of up to 10 mg of diazepam did not result in higher serum levels.

On the other hand, an earlier RCT showed that 28 days of 10 mg vaginal diazepam suppositories did not result in an improvement in high-tone pelvic floor dysfunction when compared to a placebo [15].

In addition, especially because there are several pathophysiological factors involved in the onset and progression of vulvodynia, it is advised to have a multimodal course of treatment that includes medical care, physiotherapy, and psychological care [16]. Research indicates that interdisciplinary treatment is effective in lowering pain and enhancing sexual performance in vulvodynia sufferers. Several benefits of this therapy include increased patient and healthcare provider motivation and tenacity, a multifaceted approach to vulvar pain at the same time and improved interdisciplinary coherence. According to a recent systematic review and meta-analysis, patients with vulvodynia appeared to benefit considerably more from rehabilitative techniques. More specifically, extracorporeal shockwave therapy and acupuncture demonstrated a strong improvement in pain outcomes and could be used as a first-line therapy for vulvodynia in the field of rehabilitation [17].

Experts have emphasized that vulvodynia therapy needs to be tailored to each patient [18,19]. The largest obstacle is starting a pelvic floor rehabilitation program in order to maximize the results that have an impact on patients’ well-being. In fact, vulvodynia’s psychological stress has a detrimental effect on quality of life, since women frequently report experiencing emotions of humiliation, inadequacy, emotional detachment, and altered body image. The idea of a pelvic floor rehabilitation program has a number of drawbacks. Emphasis should be placed on the patients’ reluctance, the dearth of specific information regarding methods/tools for diagnosis, treatment and follow-up, the lack of knowledge regarding the diagnosis and potential course of treatment for these conditions, and the fact that written instructions in medical reports rarely translate into recommendations to perform pelvic training.

Giving patients knowledge and support in this little-known region is crucial. It explains the importance of the pelvic floor structure in rehabilitation and how this synergistic program is a new development from the pelvic area’s point of view. It accomplishes this by highlighting the importance of the pelvic floor structure in day-to-day activities, sexual activity, and physical and mental health, and by increasing feelings of satisfaction and self-worth.

The correct communication and interpretation of information between the medical staff and patients are the most important aspects of ensuring program compliance. It should be emphasized that, in order for pelvic rehabilitation to be effective, the patient must become aware of and become proficient in the various motor abilities of the pelvic floor muscular area in order to identify the perineum’s muscles and train those muscles. It should be stressed that, for the effectiveness of pelvic rehabilitation, the patient must develop awareness and master the various motor skills of the pelvic floor muscle area to recognize the muscles of the perineum; train the muscles of the perineum by modifying the altered pelvic floor muscle parameters caused by hypoactivity, hyperactivity, or impaired coordination in functional activities; and learn to use the muscles of the perineum by transferring the exercise to different situations of daily life, improving tone muscle and pelvic microcirculation and reducing sexual discomfort [18,19].

The current study is highly applicable since it presents a treatment plan that significantly improves hypertonicity as measured by pelvic floor muscle biometry. There exist certain limitations to the proposed study. Firstly, the results obtained may have been influenced by the limited sample size and the absence of consideration of their demographic characteristics. Despite being randomized, this study’s non-blind design raises the possibility of bias, even if the experimenters and participants in the static examination had no influence over the data processing. In addition, the proposed stringent exclusion criteria may have influenced the sample size selection, and this may become a possible bias in result interpretation. Additionally, we did not do a questionnaire on sexual pleasure and everyday activities to assess the significance of physical and vaginal training in the clinical outcome measures. Instead, we just assessed the Marinoff and VAS scales. However, it is noteworthy that, despite all the critical issues, all the data analyzed came back statistically significant, so that the small variations recorded correspond to a good clinical impact. Moreover, research evaluating biometry alterations during follow-up must be promoted in order to enhance the comprehension of muscle behavior following treatment and its association with clinical outcomes.

## 5. Conclusions

The therapeutic protocol that combines intravaginal diazepam with pelvic floor rehabilitation appears promising in the treatment of vulvodynia, given the biometric and clinical improvements it entails, as well as represents an excellent tool for working on patient compliance. The hope is that further studies will be conducted to confirm these results.

## Figures and Tables

**Table 1:** Pelvic floor ultrasound characteristics before and after treatment.

	Time	Case Arm Mean (Standard Deviation)	Control Arm Mean (Standard Deviation)
Symphysis-elevator distance	Before treatment	3.9 (0.3)	3.9 (0.4)
	6 months	4 (0.3)	3.5 (0.4)
*p*-value for variation		0.23	0.01
Anorectal angle	Before treatment	122.6 (2.9)	122.4 (2.4)
	6 months	123.7 (3.1)	121.4 (2.3)
*p*-value for variation		0.28	0.01
Elevator plate angle	Before treatment	9 (2.3)	8.7 (2.5)
	6 months	9.4 (2.3)	8.2 (2.4)
*p*-value for variation		<0.01	0.01
Hiatal area diameter	Before treatment	1.3 (0.4)	1.3 (0.4)
	6 months	1.4 (0.5)	1.1 (0.4)
*p*-value for variation		<0.01	0.01
Marinoff scale	Before treatment	2.3 (0.5)	2.4 (0.5)
	6 months	1.4 (0.7)	3.4 (1.2)
*p*-value for variation		0.01	0.03
VAS scale	Before treatment	5.8 (1)	5.5 (1.3)
	6 months	2.8 (0.9)	6.4 (1)
*p*-value for variation		0.01	0.06

Legend: VAS = visual analogue scale.

**Table 2:** Difference in variations between case and control groups.

	Case Arm Mean (Standard Deviation)	Control Arm Mean (Standard Deviation)	*p*-Value
Symphysis-elevator distance	0.1 (0.2)	−0.3 (0.1)	<0.01
Anorectal angle	1 (2.6)	−1 (0.7)	0.1
Elevator plate angle	0.4 (0.3)	−0.5 (0.3)	<0.01
Hiatal area diameter	0.1 (0.1)	−0.2 (0.2)	<0.01
Marinoff scale	−0.9 (0.9)	1 (1.2)	<0.01
VAS scale	−3 (0.8)	0.9 (1.1)	<0.01

Legend: VAS = visual analogue scale.

**Table 3:** Unadjusted and adjusted models for the relationship between treatment and Marinoff score.

Unadjusted Model			
	Estimate	SE	*p*-value
Treatment	−3.9	0.4	<0.01
Adjusted model			
	Estimate	SE	*p*-value
Treatment	−3.8	0.3	<0.01
Symphysis-elevator distance	−2.6	0.7	<0.01
Anorectal angle	−0.1	0.1	0.54
Elevator plate angle	−0.2	0.1	0.02
Hiatal area diameter	3.4	0.6	<0.01

Legend: SE = standard error.

**Table 4:** Unadjusted and adjusted models for the relationship between treatment and VAS.

Unadjusted Model			
	Estimate	SE	*p*-value
Treatment	−1.9	0.5	<0.01
Adjusted model			
	Estimate	SE	*p*-value
Treatment	−1.9	0.5	<0.01
Symphysis-elevator distance	1.1	1.5	0.46
Anorectal angle	0	0.2	0.92
Elevator plate angle	−0.1	0.2	0.55
Hiatal area diameter	0.5	1.1	0.66

Legend: SE = standard error.

## Data Availability

The data that support the findings of this study are available from the corresponding author, [M.D.], upon reasonable request.
